# A Six-Month Prospective Audit of Hypoglycemia in Non-Critically Ill Inpatients at a Tertiary Care Hospital in North India: Prevalence, Presentation, and Prevention

**DOI:** 10.7759/cureus.74629

**Published:** 2024-11-27

**Authors:** Akshayaa Kumar Aggarwal, Rashmi Gupta Bajpai

**Affiliations:** 1 Medicine, SGT Medical College, Hospital and Research Institute, Gurugram, IND

**Keywords:** glycemic control, hyperglycemia, hypoglycemia, undiagnosed, unnoticed medication

## Abstract

Objective: This research aimed to assess the prevalence, presentation, and risk factors associated with hypoglycemia in non-critically ill vs. critically ill inpatients at a tertiary care hospital in North India, focusing on identifying differences in clinical parameters and outcomes between these two patient populations over six months.

Methodology: This six-month prospective study, conducted at a tertiary care hospital in North India, evaluated the frequency, presentation, and prevention of hypoglycemia in 200 hospitalized patients, evenly divided between non-critically ill and critically ill groups. Data collection involved recording baseline parameters and daily blood glucose levels and documenting hypoglycemic episodes and their severity. Preventive strategies, including glucose monitoring, medication adjustments, and dietary interventions, were also tracked. The study used chi-square and t test analysis to determine the prevalence of hypoglycemia, recurrent episodes, and the effectiveness of preventive measures, focusing on differences between the two patient groups and the impact of management strategies.

Results: The study found that critically ill patients were older (65 ± 15 years) than non-critically ill patients (60 ± 12 years, p = 0.036) with a similar proportion of females in both groups (50% vs. 45%, p = 0.527). Hypoglycemia was more common in critically ill patients (45% vs. 25%, p = 0.005), as were cardiovascular disease (50% vs. 30%, p = 0.004) and chronic kidney disease (35% vs. 20%, p = 0.023). Nutrition consultations were more frequent in non-critically ill patients (30% vs. 15%, p = 0.025), while medication adjustments were more common in critically ill patients (40% vs. 20%, p = 0.004). Non-recurrent hypoglycemia was higher in non-critically ill patients (68% vs. 44.4%, p = 0.038), whereas recurrence was higher in critically ill patients (55.6% vs. 32%, p = 0.038).

Conclusion: The study highlights the significance of addressing hypoglycemia in non-critically ill inpatients, a group that is often overlooked compared to critically ill patients. Although non-critically ill patients had fewer comorbidities and a lower incidence of previous hypoglycemia, the occurrence of hypoglycemia in this group remains a concern. The findings indicate that, even in non-critically ill patients, careful management of factors such as insulin therapy and underlying conditions like type 2 diabetes is essential to prevent hypoglycemic episodes. These results emphasize the need for targeted interventions in non-critical care settings to mitigate the risk of hypoglycemia and enhance patient safety and outcomes.

## Introduction

Hypoglycemia, characterized by low blood glucose levels, is a significant clinical concern and a common complication among hospitalized patients, particularly those with diabetes [1,2]. While hyperglycemia management has historically received considerable attention in healthcare, hypoglycemia presents substantial risks and challenges that necessitate closer examination and proactive intervention [3,4]. Despite its severity, hypoglycemia in hospitalized patients is frequently underdiagnosed and under-researched, especially among non-critically ill populations. This gap in the literature underscores the importance of comprehensively investigating the prevalence, clinical presentation, and associated risk factors of hypoglycemia in diverse patient populations. Previous studies have identified various factors contributing to hypoglycemia in hospitalized patients, including medication errors, irregular meal schedules, inadequate blood glucose monitoring, and ineffective communication among healthcare providers [5-8].

Furthermore, underlying comorbidities such as renal or hepatic impairment further complicate glycemic control, thereby increasing the risk of hypoglycemia [6,7,9]. Despite these insights, limited data are available regarding the frequency, severity, and outcomes of hypoglycemia in non-critically ill inpatients. This population often receives less attention compared to critically ill patients.

This study seeks to address these gaps by evaluating the prevalence of hypoglycemia in critically ill and non-critically ill inpatients at a tertiary care hospital in North India, analyzing differences in clinical presentation and severity of hypoglycemia between these groups, and identifying key risk factors, including patient-specific and treatment-related variables. Additionally, it examines the effectiveness of current prevention strategies in minimizing hypoglycemic episodes. By focusing on the non-critically ill inpatient population-an understudied group in hypoglycemia research-this study provides novel insights into their unique risk profiles and management challenges. The findings aim to fill a critical gap in the literature by informing targeted interventions to reduce hypoglycemia recurrence, improve patient safety, and optimize glycemic control, thereby contributing to improved outcomes and quality of care for hospitalized patients.

## Materials and methods

Study design

This prospective study was conducted over six months (from January 2023 to June 2023) at a tertiary-care hospital in North India. The primary objective was to evaluate the frequency, presentation, and prevention of hypoglycemia in both non-critically ill and critically ill patients. The study included 200 participants admitted to various medical and surgical wards, including ICUs. Participants were categorized into two groups: non-critically ill (n = 100) and critically ill (n = 100) hospitalized patients.

Sample population and inclusion criteria

The sample population consisted of adult patients aged 18 years or older who were hospitalized with a confirmed diagnosis of diabetes mellitus or were at risk of hypoglycemia due to medical conditions or medication use. Non-critically ill patients included those admitted to general medical or surgical wards with non-life-threatening conditions. In contrast, critically ill patients required admission to ICUs for severe illnesses necessitating intensive monitoring and treatment. Patients under the age of 18, those diagnosed with type 1 diabetes, pregnant individuals, and those discharged within 24 hours of admission were excluded from the study.

The sample size of 200 participants, with 100 in each group, was determined using a power analysis to ensure sufficient statistical power to detect significant differences between the two groups. The study assumed an anticipated hypoglycemia incidence of 30% in critically ill patients and 15% in non-critically ill patients, with a significance level of 0.05 and a power of 0.80. This calculation yielded a requirement of approximately 90 participants per group. To account for potential dropouts or incomplete data, the sample size was increased to 100 participants per group, ensuring robustness in detecting statistically significant outcome differences.

Data collection

Data were collected using structured case report forms, with baseline parameters such as age, gender, BMI, comorbidities (e.g., type 2 diabetes, chronic kidney disease, cardiovascular disease), and history of hypoglycemia recorded upon admission. Risk factors, including the use of insulin therapy, were also documented. Hypoglycemia was defined as a blood glucose level below 70 mg/dL, measured using point-of-care glucose meters or confirmed by laboratory venous blood glucose readings. The clinical manifestations of hypoglycemia were systematically categorized into asymptomatic, mild, or severe episodes based on established guidelines.

Asymptomatic hypoglycemia was defined as a documented low blood glucose level without accompanying symptoms. Mild hypoglycemia included symptoms such as sweating, dizziness, or hunger that could be self-managed without external assistance. Severe hypoglycemia encompassed episodes requiring external assistance, often due to severe symptoms such as confusion, seizures, or unconsciousness. These classifications were consistent with established standards in diabetes care, ensuring uniformity in defining and recording hypoglycemic events.

Prevention and management strategies

Preventive measures to minimize the occurrence of hypoglycemia were systematically recorded. These included regular blood glucose monitoring, adjustments to antidiabetic medications, and dietary interventions. Dietary interventions involved individualized nutrition counseling to ensure consistent carbohydrate intake and meal timing. Patients were advised on small, frequent meals to prevent glucose fluctuations, and those receiving enteral or parenteral nutrition underwent additional glucose monitoring to optimize caloric and macronutrient intake and reduce the risk of hypoglycemia.

Medication adjustments, particularly insulin and oral hypoglycemic agents, were made based on standardized protocols. Insulin doses were reviewed and adjusted daily for critically ill patients and every 48 to 72 hours for non-critically ill patients, or more frequently if clinically indicated. The protocol prioritized the reduction of basal insulin doses, with correctional insulin used as needed to manage glucose levels. Oral antidiabetic agents were reviewed for dosing and timing modifications to reduce hypoglycemic risk, particularly in patients with fluctuating glucose levels or diminished oral intake.

Outcomes

The study's primary outcome was the frequency of hypoglycemic episodes during hospitalization. Secondary outcomes included the rate of recurrent hypoglycemia, defined as two or more episodes within 24 hours or during hospitalization, the clinical manifestations associated with hypoglycemia, and the effectiveness of dietary and pharmacological interventions in reducing hypoglycemic events. These outcomes were recorded in non-critically ill and critically ill patient groups, providing a comprehensive evaluation of hypoglycemia management and prevention strategies.

Quantitative data analysis

Statistical analysis was performed on the collected data by using SPSS 23.0 version. Descriptive statistics summarized baseline characteristics. Continuous variables, such as age and BMI, were presented as means and SDs, while categorical variables, such as gender and comorbidities, were reported as frequencies and percentages. The chi-square test was used to compare categorical variables between the non-critically ill and critically ill groups, while independent sample t-tests were employed for continuous variables. A p-value of less than 0.05 was considered statistically significant. The prevalence of hypoglycemia was calculated as the percentage of patients who experienced at least one episode during hospitalization. Significant differences in the presentation of hypoglycemia (asymptomatic, mild, or severe) between the groups were identified. Chi-square tests were also conducted to evaluate the effectiveness of various preventive strategies in reducing the risk of hypoglycemia and recurrent hypoglycemia.

Ethical considerations

The study protocol was reviewed and approved by the hospital’s institutional review board (IRB) (SGT/IEC/2022/990). Informed consent was obtained from all participants or their legal representatives before enrollment. Patient confidentiality was strictly maintained, and data were anonymized before analysis.

## Results

The results of the analysis comparing non-critically ill and critically ill patients are summarized in Table 1. The mean age of non-critically ill patients was 60 ± 12 years, significantly lower than the 65 ± 15 years observed in critically ill patients (p = 0.036). BMI was similar across both groups: non-critically ill patients had a BMI of 27.5 ± 4 kg/m², while critically ill patients had a BMI of 28 ± 4.5 kg/m², with no statistically significant difference (p = 0.448). There was no significant difference in sex distribution between the groups, with 45 (45%) of non-critically ill patients being female, compared to 50 (50%) of critically ill patients (p = 0.527). A history of previous hypoglycemia was more common among critically ill patients, with 30 (30%) reporting such a history compared to 15 (15%) in the non-critically ill group (p = 0.010). Type 2 diabetes mellitus was present in 70 (70%) of non-critically ill patients and 80 (80%) of critically ill patients, though this difference was not statistically significant (p = 0.096). Cardiovascular disease was significantly more prevalent in critically ill patients, affecting 50 (50%) compared to 30 (30%) of non-critically ill patients (p = 0.004). Chronic kidney disease was also more common among critically ill patients, affecting 35 (35%) vs. 20 (20%) in the non-critically ill group (p = 0.023). Lastly, insulin therapy usage was higher in critically ill patients, with 60 (60%) using insulin compared to 40 (40%) in the non-critically ill group; however, this difference approached but did not reach statistical significance (p = 0.07).

**Table 1:** Baseline characteristics and risk factors *  t test value; ** chi square p<0.05 was considered significant

Characteristics	Non-critically ill (N= 100)	Critically ill (N=100)	Statistical test	p-value
Age (in years)	60 ± 12	65 ± 15	2.12*	0.036
BMI (in kg/m^2^)	27.5 ± 4	28 ± 4.5	0.76*	0.448
Sex			0.40**	0.527
Female	45 (45%)	50 (50%)		
Male	55 (55%)	50 (50%)		
History of previous hypoglycemia	15 (15%)	30 (30%)	6.67**	0.010
Type 2 diabetes mellitus	70 (70%)	80 (80%)	2.78**	0.096
Cardiovascular disease	30 (30%)	50 (50%)	8.33**	0.004
Chronic kidney disease	20 (20%)	35 (35%)	5.13**	0.023
Use of insulin therapy	40 (40%)	60 (60%)	7.27**	0.07

The comparison of hypoglycemia prevalence and symptom severity between the two groups yielded notable findings. Hypoglycemia was significantly more prevalent among critically ill patients, affecting 45 (45%) compared to 25 (25%) in the non-critically ill cohort (p = 0.005). However, there were no statistically significant differences in the severity of hypoglycemic symptoms between the groups. Severe symptoms were reported by 5 (30%) of hypoglycemic non-critically ill patients and 10 (22.2%) of hypoglycemic critically ill patients (p = 0.82). Mild symptoms were experienced by 10 (40%) in the non-critically ill group and 20 (44.4%) in the critically ill group (p = 0.75). Asymptomatic hypoglycemia was noted in 10 (40%) of non-critically ill patients and 15 (33.3%) of critically ill patients (p = 0.61). These findings suggest that while hypoglycemia is more common in critically ill patients, the severity of symptoms were statistically insignificant between the groups (Table 2).

**Table 2:** Prevalence and presentation of hypoglycemia

Variables	Non-critically ill (N=100)	Critically ill (N=100)	Chi-square	p-value
Patients with hypoglycemia	25 (25%)	45 (45%	8	0.005
Severe symptoms	5 (30%)	10 (22.2%)	0.05	0.82
Mild symptoms	10 (40%)	20 (44.4%)	0.10	0.75
Asymptomatic	10 (40%)	15 (33.3%)	0.25	0.61

Finally, the comparison of management strategies and hypoglycemia outcomes revealed several significant differences. Non-critically ill patients received nutrition consultations and meal planning more frequently, with 30 (30%) benefiting from these services compared to 15 (15%) of critically ill patients (p = 0.025). Regular blood glucose monitoring was prevalent in both groups, with 80 (80%) of non-critically ill patients and 90 (90%) of critically ill patients participating in this practice; however, this difference was not statistically significant (p = 0.063). Adjustment of antidiabetic medication was significantly more common in the critically ill cohort, with 40 (40%) receiving adjustments compared to 20 (20%) of non-critically ill patients (p = 0.004). Regarding hypoglycemia outcomes, the non-recurrence of hypoglycemia was significantly higher in the non-critically ill group, with 17 (68%) experiencing no recurrence, compared to 20 (44.4%) of critically ill patients (p = 0.038). Conversely, recurrent hypoglycemia was more frequent in critically ill patients, affecting 25 (55.6%) compared to 8 (32%) of non-critically ill patients (p = 0.038) (Table 3).

**Table 3:** Prevention strategies and outcomes of hypoglycemia

Variables	Non-critically ill (N=100)	Critically ill (N=100)	Chi-square	p-value
Nutrition consultations and meal planning	30 (30%)	15 (15%)	5.00	0.025
Regular blood glucose monitoring	80 (80%)	90 (90%)	3.47	0.063
Adjustment of antidiabetic medication	20 (20%)	40 (40%)	8.16	0.004
No recurrence	17 (68%)	20 (44.4%)	4.31	0.038
Recurrent hypoglycemia	8 (32%)	25 (55.6%)	4.31	0.038

## Discussion

This six-month prospective study aimed to assess the frequency, presentation, and prevention strategies for hypoglycemia in both critically ill and non-critically ill patients at a tertiary care hospital in North India. The study found a significantly higher incidence of hypoglycemia in critically ill patients (45%) compared to non-critically ill patients (25%), underscoring the need for tailored management strategies for different patient populations.

The increased prevalence of hypoglycemia in critically ill patients observed in our study aligns with previous research. For instance, Ling et al. reported a 17.0% prevalence of hypoglycemia in the ICU [10]. Similarly, Wiener et al. noted a significant rise in the risk of hypoglycemia in ICUs due to stringent glucose management compared to routine care [11]. Our analysis suggests that the higher rate of hypoglycemia might be attributed to changes in ICU protocols, such as those implemented in the NICE-SUGAR trial, which linked severe hypoglycemia to stricter glucose control [12].

In contrast, the incidence of hypoglycemia among non-critically ill patients (25%) was somewhat lower but still notable. Previous studies, such as Farrokhi et al., have documented hypoglycemia rates among non-critically ill inpatients ranging from 3.5% to 16% [13]. The higher incidence observed in our study may be due to factors such as insulin therapy and concurrent conditions like chronic renal disease. This finding is consistent with the existing literature, which indicates that patients with multiple risk factors are more vulnerable to hypoglycemia.

Our investigation did not reveal any statistically significant differences in the clinical manifestations of hypoglycemia between the two groups, with comparable occurrences of asymptomatic, mild, and severe hypoglycemia. This finding contrasts with specific studies, such as the one by Finfer et al., which reported a higher incidence of severe hypoglycemia in critically ill patients [12]. However, it aligns with the results of the study by Li et al., who observed similar hypoglycemia symptoms across different settings [14]. These results suggest that severe hypoglycemia episodes can occur in both critically ill and non-critically ill patients due to underlying health issues and treatment regimens.

The study also revealed significant differences in preventive strategies. Critically ill patients had a higher prevalence of regular blood glucose monitoring (90%) compared to non-critically ill patients (80%). Additionally, critically ill patients experienced a significantly higher frequency of antidiabetic medication adjustments (40% vs. 20%). These findings are consistent with the American Diabetes Association’s (ADA's) recommendations for ICU settings, which advocate for rigorous patient monitoring and medication adjustments to prevent glucose fluctuations [15]. In contrast, non-critically ill patients received more frequent nutrition consultations and meal planning (30% vs. 15%), reflecting a more individualized approach to diabetes management. Research by Umpierrez et al. has demonstrated the effectiveness of tailored dietary interventions in reducing hypoglycemia in non-critically ill patients, which is consistent with our results [16].

Despite these preventive measures, critically ill patients had a significantly higher prevalence of recurrent hypoglycemia (55.6% vs. 32%). This finding corroborates the results of the study by Bagshaw et al., who established a link between recurrent low blood sugar in ICU patients and higher morbidity and mortality rates [17]. These results highlight the complex nature of glucose regulation in critically ill patients, where various factors contribute to the risk of hypoglycemia.

Contemporary literature emphasizes the intricate balance required in managing hypoglycemia in hospitalized patients. Studies such as by Turchin et al. and Clain et al. underscore the importance of maintaining a delicate equilibrium between the risks of hypoglycemia and strict glucose control, particularly in ICU environments [18,19]. Yao et al. conducted a study revealing the potential of continuous glucose monitoring (CGM) devices to reduce severe hypoglycemia episodes in ICU patients, suggesting that CGM is a valuable tool for managing high-risk patients effectively [20]. The ADA and the Society of Critical Care Medicine (SCCM) now recommend personalized glucose targets to manage both hypoglycemia and hyperglycemia risks effectively [21]. These guidelines are consistent with our findings and highlight the need for ongoing research to improve hypoglycemia management.

Strength and limitations of the study

The strengths of this study lie in its comprehensive approach to evaluating hypoglycemia in diverse inpatient populations, including both non-critically ill and critically ill patients. By examining hypoglycemia across these settings, the study provides valuable insights into differences in presentation, management, and outcomes between these groups. Furthermore, the prospective design ensures the systematic and real-time collection of data, reducing the risk of recall bias and enhancing the reliability of the findings. This methodological rigor allows for a detailed analysis of hypoglycemic episodes, their severity, and the effectiveness of preventive strategies, contributing to a deeper understanding of hypoglycemia management in hospitalized patients.

However, it is essential to acknowledge the limitations of this study, including its single-center design, which may limit the generalizability of the findings to other healthcare settings with different patient populations and resource availability. The relatively small sample size may also reduce the statistical power to detect certain differences or associations. Additionally, the study did not assess long-term outcomes, preventing an understanding of the prolonged impact of hypoglycemia management strategies on patient health. The reliance on intermittent glucose monitoring, rather than CGM, may have led to missed episodes of asymptomatic hypoglycemia. Finally, the study did not account for variations in clinical practices or protocols between different wards, which could have influenced the results. Future research should prioritize multicenter trials with larger, more diverse populations and incorporate advanced technologies, such as CGM, to improve hypoglycemia detection and management while assessing long-term outcomes.

## Conclusions

The study highlights the significance of addressing hypoglycemia in non-critically ill inpatients, a group that is often overlooked compared to critically ill patients. While non-critically ill patients had fewer comorbidities and a lower incidence of previous hypoglycemia, the occurrence of hypoglycemia in this group remains a concern. The findings underscore the importance of carefully managing factors such as insulin therapy and underlying conditions like type 2 diabetes to prevent hypoglycemic episodes in this population.

However, the generalizability of these results to other hospitals or healthcare systems may be limited due to the study’s single-center design and relatively small sample size. Multicenter studies or research involving larger and more diverse populations are needed to confirm these findings and explore outcome variations across different settings. Additionally, further research is recommended to investigate the causal impact of specific interventions, such as individualized dietary modifications and standardized medication adjustment protocols, on hypoglycemia prevention. These efforts will help develop evidence-based strategies to enhance patient safety and outcomes in critical and non-critical care settings.
